# Transgenic expression of a functional fragment of harpin protein Hpa1 in wheat induces the phloem-based defence against English grain aphid

**DOI:** 10.1093/jxb/ert488

**Published:** 2014-03-25

**Authors:** Maoqiang Fu, Manyu Xu, Ting Zhou, Defu Wang, Shan Tian, Liping Han, Hansong Dong, Chunling Zhang

**Affiliations:** State Ministry of Education Key Laboratory of Integrated Management of Crop Pathogens and Insect Pests, Nanjing Agricultural University, Nanjing, 210095, China

**Keywords:** Agronomic traits, English grain aphid, ethylene signalling, Hpa110–42 expression, insect defence, phloem-based defence, transgenic wheat.

## Abstract

The harpin protein Hpa1 has multiple beneficial effects in plants, promoting plant growth and development, increasing crop yield, and inducing resistance to pathogens and insect pests. For these effects, the 10–40 residue fragment (Hpa1_10–42_) isolated from the Hpa1 sequence is 1.3- to 7.5-fold more effective than the full-length protein. Here it is reported that the expression of Hpa1_10–42_ under the direction of an insect-induced promoter induces the phloem-based defence to English grain aphid, a dominant species of wheat aphids. The expression of Hpa1_10–42_ was found to compromise the colonization preference of aphids on the plant and further inhibit aphid reproduction in leaf colonies. In Hpa1_10–42_-expressing wheat lines, moreover, aphid feeding from the phloem was repressed in correlation with the phloem-based defence. This defensive mechanism was shown as enhanced expression of wheat genes encoding phloem lectin proteins (PP2-A1 and PP2-A2) and β-1,3-glucan synthase-like enzymes (GSL2, GSL10, and GSL12). Both PP2-A and β-1,3-glucan formed high molecular mass polymers to block phloem sieve plate pores and therefore impede aphid feeding from the phloem. However, the phloem-based defence was impaired by treating plants with ethylene signalling inhibitors, suggesting the requirement for the ethylene signalling pathway. In addition, if Hpa1_10–42_-expressing plants were subjected to attack by a small number of aphids, they newly acquired agriculturally beneficial characters, such as enhanced vegetative growth and increased tiller numbers and grain output values. These results suggest that the defensive and developmental roles of Hpa1_10–42_ can be integrated into the germplasm of this agriculturally significant crop.

## Summary

A 33 amino acid fragment sequence of 136 residue Hpa1 harpin expressed in transgenic wheat induces the phloem defence that efectively represses infestations of English grain aphid on the crop.

## Introduction

Hpa1 (synonym HpaG) is a harpin protein produced by *Xanthomonas oryzae*, an important bacterial pathogen of rice (Zhu *et al.*, 2000). Like all harpin orthologues identified in different species of Gram-negative plant pathogenic bacteria (Wei *et al.*, 1992; He *et al.*, 1993; Dong *et al.*, 1999; Kim and Beer, 2000; Liu *et al.*, 2006), Hpa1 induces plant growth and defence responses (Peng *et al.*, 2004; Liu *et al.*, 2006; Ren *et al.*, 2006*a*, *b*; Wu *et al.*, 2007; C. Zhang *et al.*, 2007, 2011; Chen *et al.*, 2008*a*, *b*; Sang *et al.*, 2012). The dual effect depends on plant sensing of the N-terminal region in the Hpa1 sequence (Wang *et al.*, 2008; Li *et al.*, 2013*a*). From this region, the 10–42 residue fragment (Hpa1_10–42_) was isolated, produced by prokaryotic expression (Wu *et al.*, 2007; Chen *et al.*, 2008*a*; Li *et al.*, 2013*a*), and analysed for its effects on *Arabidopsis* (biological model plant), tobacco (cash crop), tea (drinking crop), and rice (food crop). In these plants, Hpa1_10–42_ is 1.3- to 7.5-fold more effective than the full-length Hpa1 in inducing resistance to pathogens and enhancing plant growth or increasing crop products (Wu *et al.*, 2007; Chen *et al.*, 2008*a*, *b*; Li *et al.*, 2013*a*). In tea plants, Hpa1_10–42_ is 1.3-fold more active than Hpa1 in elevating the yield of the top three leaves used as drinking material (Wu *et al.*, 2007). In rice, Hpa1_10–42_ is 2.7 and 7.5 times stronger than Hpa1 in eliciting resistance to blast (Chen *et al.*, 2008*b*) and bacterial blight (Chen *et al.*, 2008*a*). The growth enhancement is 1.5-fold higher (Chen *et al.*, 2008*a*) and the grain yield increase is 2.0-fold more (Chen *et al.*, 2008*b*) in rice plants treated with Hpa1_10–42_ compared with Hpa1. In tobacco, however, Hpa1_10–42_ is nearly 30-fold less active than Hpa1 in eliciting hypersensitive cell death (HCD) (Chen *et al.*, 2008*a*). HCD is a defence response and also a developmental cost associated with defence responses (Dangl *et al.*, 1996; Yu *et al.*, 1998; Peng *et al.*, 2004). Indeed, resistance is activated in an HCD-independent manner in Hpa1-expressing transgenic tobacco (Peng *et al.*, 2004). Therefore, Hpa1_10–42_ is a desired agricultural agent that induces plant growth enhancement and defence responses with little cost to plant development (Peng *et al.*, 2004; Wu *et al.*, 2007; Chen *et al.*, 2008*a*, *b*).

One of the multiple effects of harpins in plants is to induce resistance to insects, especially aphids (Dong *et al.*, 2004; Liu *et al.*, 2010; Lü *et al.*, 2011, 2013; C. Zhang *et al.*, 2011). Aphids represent a typical group of phloem-feeding insects that are highly specialized in their mode of feeding (Tjallingii, 1988, 2006; Tjallingii and Esch, 1993) and produce a unique stress on plant fitness (Will and van Bel, 2006, 2008; De Vos and Jander, 2009). The stress is often devastating to the production of agriculturally significant crops, such as wheat (*Triticum aestivum* L.). Wheat aphids mainly belong to *Schizaphis graminum* Rondani, *Rhopalosiphum padi* Linnaeus, and *Sitobion avenae* Fabricius (Basky and Fónagy, 2003). These species are indigenous, and *S. avenae* (commonly called English grain aphid) is dominant in China (Hong and Ding, 2007). Aphids attack every aerial part of wheat during the plant’s development from Feekes stage 1 (one-shoot stage) through to Feekes stage 11 (grain-ripening stage) (Nelson *et al.*, 1988). Aphid attacks cause chlorosis and necrosis with repression of photosynthesis in aerial organs of wheat, or cause direct damage to wheat grains, resulting in a severe decrease in the grain yield (Hong and Ding, 2007). Aphids have strong capabilities for multiplication and constantly attack plants with huge populations, which pose challenges for insect management. If a harpin induces growth and defence in wheat as in other plants, the dual effect may compensate for aphid-induced damage and contribute to effective control of the insect.

The multiple effects of harpins are attributable to cross-talk of distinct hormone signalling pathways that regulate development and defence in plants (Chen *et al.*, 2008*a*). Harpin-induced plant growth and resistance to a phloem-feeding generalist, the green peach aphid (*Myzus persicae* Sulzer), is coordinated by the ethylene signalling pathway in *Arabidopsis* (Dong *et al.*, 2004; Lü *et al.*, 2011, 2013). In response to a harpin protein, the ethylene signalling regulators EIN5 and EIN2 act to confer growth and resistance phenotypes, respectively (Dong *et al.*, 2004). Also, in response to harpin, the ethylene signalling pathway recruits the transcription factor MYB44 to co-regulate the phloem-based defence, which specifically resists attacks by phloem-feeding insects (Liu *et al.*, 2010; Lü *et al.*, 2011, 2013; C. Zhang *et al.*, 2011). Expression of the *MYB44* gene is induced by aphid infestations or by ethylene, either applied to plants or produced in harpin-treated plants (Liu *et al.*, 2010, 2011). The 3′-terminal 2000 nucleotide fragment (*44P*_*2000*_) isolated from the predicted 3500 nucleotide sequence of the *MYB44* gene promoter is sufficient to direct *MYB44* transcription in response to ethylene or a harpin protein (Liu *et al.*, 2010, 2011). The *44P*_*2000*_-controlled expression of *MYB44* leads to the production of the MYB44 protein and its localization to the nucleus. Inside the nucleus, MYB44 binds to the promoter of *EIN2* and activates its transcription. In the presence of ethylene, moreover, the EIN2 protein exists stably in the cytosol to perform multiple roles in plant development and defence (Alonso *et al.*, 1999; Wang *et al.*, 2009; Qiao *et al.*, 2009, 2012).

One of the roles that EIN2 plays is to cooperate with MYB44 in regulating the phloem-based defence in *Arabidopsis* (Lü *et al.*, 2011; C. Zhang *et al.*, 2011). The defence essentially involves synchronized expression of the *PP2-A* gene, which encodes the phloem lectin protein PP2-A (C. Zhang *et al.*, 2011), and the *GSL5* gene, which encodes the β-1,3-glucan synthase GSL5 (Lü *et al.*, 2011). Subsequently, the PP2-A protein dimerizes and the dimer is further linked with phloem protein PP1 to form a high molecular weight polymer that accumulates to block phloem sieve plate pores (Read *et al.*, 1983; Dinant *et al.*, 2003; Kehr, 2006; Will *et al.*, 2006; Beneteau *et al.*, 2010). This process accompanies the biosynthesis of β-1,3-glucan callose via catalysis by the synthase and subsequent coagulation on sieve plates and closure of sieve plate pores (Stone and Clarke, 1992; Lü *et al.*, 2011, 2013). In harpin-treated plants, the GLS5-mediated callose coagulation on sieve plates and the closure of sieve plate pores by callose and AtPP2–PP1 complexes impede the phloem-feeding activity of the green peach aphid (Lü *et al.*, 2011, 2013). Therefore, PP2-A and GSL5 are indispensable components of the phloem-based defence that is inducible by harpin and regulated by EIN2 and MYB44. In addition, MYB44 is implicated in salicylic acid signalling for resistance to pathogens (Jung *et al.*, 2010; Zou *et al.*, 2013) and abscisic acid signalling for drought tolerance (Jung *et al.*, 2008), while the induction of both signalling pathways is a conserved function of harpin (Dong *et al.*, 2005; Zhang *et al.*, 2007; Ren *et al.*, 2008). These findings suggest that MYB44 is an integrator of harpin-activated development and defence pathways.

To integrate the developmental and defensive roles of Hpa1 and MYB44 into germplasm of an agriculturally significant crop, a cultivar of common wheat was transformed with a genetic recombinant made of *44P*_*2000*_ and the Hpa1_10–42_-coding sequence (Chen *et al.*, 2008*a*; Li *et al.*, 2013*b*). It was postulated that the robust roles of Hpa1_10–42_ in plant development and defences observed previously (Wu *et al.*, 2007; Chen *et al.*, 2008*a*, *b*) could be performed in transgenic wheat lines. In support of this hypothesis, Hpa1_10–42_ expressed in transgenic wheat lines is able to induce defence responses and enhance resistance to the scab disease (Li *et al.*, 2013*b*; Yang *et al.*, 2013). Here, it is shown that Hpa1_10–42_ expression induces the phloem-based defence against English grain aphid, a dominant species of wheat aphids.

## Materials and methods

### Plant material and growth conditions

The initial material for transformation was Yangmai16 (Y16), a wheat variety widely planted in the East China wheat-producing area. Y16 seeds were provided by Dr Yong Zhang (Academy of Agricultural Sciences of Yangzhou City, Jiangsu Province, China). T_3_ progeny of Y16:Hpa1_10–42_ lines (Li *et al.*, 2013*b*) were used in this study and their seeds were maintained in the lab. For use in surveys of plant growth and development traits, seeds were grown in 15 litre pots containing the natural loam from a wheat field near Pailou Village, Xuanwu District, Nanjing City, Jiangsu Province. Seeds in pots were germinated and plants were grown under controlled temperature (21–25 °C) and natural light conditions in a glass-equipped greenhouse affiliated to Nanjing Agricultural University and located at Pailou Village. Fertilization, irrigation, and other agronomic management were performed regularly as in the field. For use in monitoring of aphid feeding activities, plants were grown in 12cm pots, one plant per pot, in a chamber under 22 °C, 250 μE m^–2^ s^–1^ illumination, and short day (12h light/12h dark) conditions. Plants grown in the greenhouse and chamber were used in different experiments 30 d after planting, unless otherwise specified.

### Plant gene expression analysis

For use in gene expression analysis, total RNA was isolated from the top first and second expanded leaves and subjected to real-time reverse transcription–PCR (RT–PCR) using the constitutively expressed *Actin* gene as a reference (Chen *et al.*, 2008*a*; Liu *et al.*, 2010). Specific primers are provided in Supplementary Table S1 available at *JXB* online. Genes were amplified for <26 cycles with a range of template concentrations increasing by 0.5ng from 0 to 3.0ng in 25 μl reaction solutions to select the desired doses. Reaction treatments, RT–PCR protocols, product cloning, and sequencing verification were performed as previously described (Chen *et al.*, 2008*a*; Liu *et al.*, 2011). The 25 μl reaction mixture was composed of 1 μl of first-strand cDNA diluted 1:10, 2.5 μM primer, and 1 × SYBR Premix Ex Taq (TaKaRa Biotech. Co., Ltd, Dalian, China). All reactions were performed in triplicate with null-template controls in which cDNA was absent. Average expression levels of the tested genes were normalized to the null-template controls and quantified relative to *Actin1*.

### Aphid culture

A single isolate of English grain aphid was collected from the field-grown Y16 plants near Nanjing in China. A clone of apterous agamic females was obtained by acclimatization in Y16 grown in the chamber (22 °C; 250 μE m^–2^ s^–1^; short day). The colony was maintained in nursery Y16 seedlings and was transferred to fresh plants every 2 weeks. Uniform 10-day-old aphids were used in this study and were transferred to experimental plants with a fine paintbrush.

### Plant colonization

Five plants of a Y16:Hpa1_10–42_ line were interplanted with five plants of Y16 grown in the same pot for 30 d before colonization with aphids. Uniform 10-day-old aphids were placed on the upper sides of the top two expanded leaves; 10 aphids per leaf. A total of 600 aphids were monitored in three repetitions of the experiments for each genotype of plant. In each experimental repetition, 200 aphids were placed on 20 leaves of 10 plants. In the subsequent 5 d, aphid movement was monitored every 2h, and the number of aphids in each leaf colony was scored. Plant genotype preference was quantified based on the number of aphids that remained in the original leaf colony, or, conversely, the number of aphids that moved from the original colony and relocalized on leaves of Y16 or different genotypes (Y16:Hpa1_10–42_ lines). Relocalized aphids were removed immediately to avoid interfering with reproduction surveys. For reproduction, newborn nymphs were counted and then removed twice a day. The reproduction rate was quantified as the ratio between the total numbers of nymphs produced in 5 d and the total numbers of aphid adults that stayed in their original leaf colonies during the same period.

### Monitoring of aphid feeding behaviour

Aphid feeding activities were analysed by the electrical penetration graph (EPG) technique using the Giga-8 EPG system (Giga-4/8 EPG systems, Dr WF Tjallingii, Wageningen, The Netherlands). Uniform nymphs at the second instar were placed on the upper side of the top first expanded leaves of different wheat genotypes (Y16 or Y16:Hpa1_10–42_ lines). For each genotype of plant, 40 aphids placed on five plants were monitored in five repetitions of experiments. Immediately after aphids were placed on leaves, a 20mm diameter gold wire was attached to the dorsal surface of each aphid’s abdomen using silver conductive paint. The other end of the wire was connected to an eight-channel Giga-8 direct current amplifier with four channels and 10^9^ Ω input resistance in an electrical circuit that is also connected to the plant via an electrode placed in the soil. The behaviour of individual aphids was monitored for 6h. Voltage waveforms were digitized at 100 Hz with an A/D converter USB device. Waveform patterns were identified according to previously described categories (Tjallingii and Esch, 1993; C. Zhang *et al.*, 2011). Waveform recordings were dissected each 5 s with the EPG analysis software Stylet^+^ (EPG system, Wageningen, The Netherlands; www.epgsystems.eu) installed on a computer connected to a Giga-8 direct current amplifier.

### Callose visualization

Callose deposition in leaves was determined using a previously described protocol (Lü *et al.*, 2011). The top two leaves were infiltrated with 5ml of a solution made up of phenol, glycerol, lactic acid, water, and 95% ethanol (1:1:1:1:2, v/v/v/v). Leaves in solution were incubated in a 65 °C bath until they were judged clear and then were stained with aniline blue. The staining reaction was left in the dark for 4h. Leaf samples were observed by microscopy under an ultraviolet field, and callose deposition in vascular bundles of leaf middle veins leaves was visualized as a blue colour.

### Pharmacological study

Plant treatments with AgNO_3_ or 1-methylcyclopropene (1-MCP) were performed as previously described (Dong *et al.*, 2004; Zhang *et al.*, 2007; Ren *et al.*, 2008). An aqueous solution of 20 μM AgNO3 was freshly prepared before use and amended with 0.03% (v/v) Silwet-37 as a surfactant, and the mixture was applied to plants by spraying over plant tops. Plants were treated similarly with 0.03% Silwet-37 in the experimental control group. Use of water-volatilizable 1-MCP tablets (Lytone Enterprise Inc., Nanjing Agency) was according to the vendor’s protocol. Immediately before treatment, tablets were volatilized in water in a small beaker to release gaseous 1-MCP into plants growing in pots. The pots were placed together with the beaker in a 12cm^3^ glass box which was immediately sealed. The 1-MCP gas was adjusted to a final concentration of 0.22 μl l^–1^ by using the correct amounts of the tablets. Plants were treated in this way for 6h. In the experimental control group, plants were incubated similarly but 1-MCP was not applied. In both pharmacological treatments, plants were colonized with aphids, the phloem-based defences were analysed 6h later, and aphid colonization and feeding activities were investigated after an additional 18h as described above.

### Plant growth and grain analyses

Plants grown in the greenhouse were divided into two experimental groups. In the first group, plants were prevented from any aphid infestations throughout the life cycle until seed harvest. In the second group, plants were colonized with the second instar nymphs of English grain aphid. The artificial colonization was performed three times, 10 nymphs per leaf each time; nymphs were placed on growing leaves of 10-day-old seedlings, and then placed on the top first and second expanded leaves at littering and flowering stages. In both experimental groups, the vegetative growth was evaluated by the number of tillers per plant and the fresh weight of plants was determined when the first heads were visible. After harvest, grain characters were analysed by the machine version method (Majumdar and Jayas, 2000) using an SC-I Colored and Automatic Seed Analyzer (Visual Detection Institute, Zhejiang Sci-Tech University, Hangzhou, China). Root growth and branching were assessed in independent experiments. Plants were grown in loam in pots or in a nutrient solution (Tocquin *et al.*, 2003) in plastic tubes (2cm in diameter and 18cm tall). Plants in pots were grown in the greenhouse and plants in tubes were grown in the chamber. In both cases, plants remained free from aphids or 10-day-old plants were colonized with aphid nymphs as stated above. Roots of 25-day-old plants were observed, root branches were counted, and the length of every branch was scored.

### Statistical analysis

Statistical analysis was performed to compare differences in tested characters among the Y16 plant and Y16:Hpa1_10–42_ lines or among different treatments (including control) in the pharmacological study. The IBM SPSS19.0 software package (IBM Corporation, Armonk, NY, USA; http://www-01.ibm.com/software/analytics/spss/) was used according to instructions in a text book that describes in detail analysis methods using IBM SPSS19.0 (Shi, 2012). Statistic homogeneity of variance in data was determined by Levene test, and the statistically formal distribution pattern of the data was confirmed by the P-P Plots program. Then, data were analysed by analysis of variance (ANOVA) together with Fisher’s least significant difference (LSD) test.

## Results

### Hpa1_10–42_ expression is induced by aphid infestation in transgenic wheat lines

**Fig. 1.**
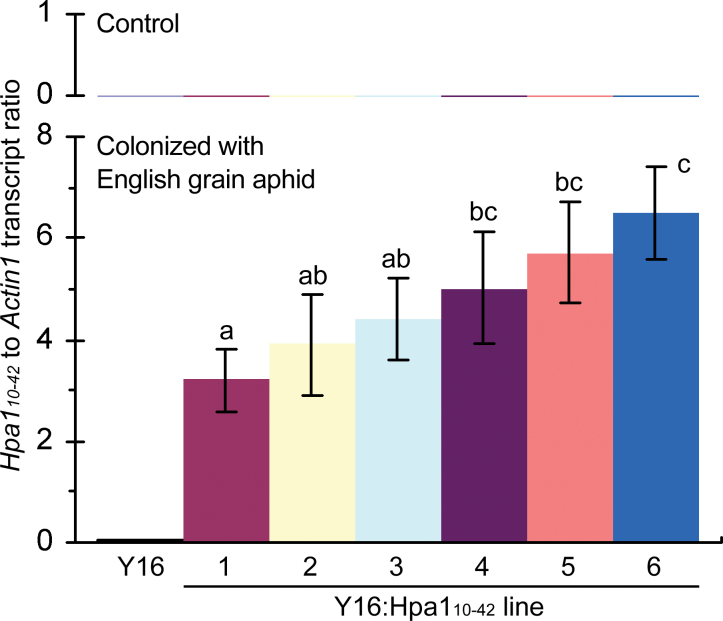
Analysis of *Hpa1*_*10–42*_ expression in transgenic wheat lines (Y16:Hpa1_10–42_#1 to #6) in comparison with Y16 used as a transformation initial parent cultivar of wheat. Real-time reverse transcription–PCR (RT–PCR) analysis using the constitutively expressed *Actin1* gene as a reference. RNA used in the analysis was isolated from leaves of plants that had been colonized with aphids or not colonized in the control. The *Hpa1*_*10–42*_/*Actin1* transcript ratio is the mean value ±SD of results from three experimental repeats (15 plants/repeat). Different letters on the SD bars indicate significant differences among compared plants by the one-tailed ANOVA method and Fisher’s LSD test (*P*<0.01). (This figure is available in colour at *JXB* online.)

Transformation of the wheat cultivar Y16 with a genetic recombinant that contained the functional fragment *44P*_*2000*_ of the *MYB44* gene promoter (Liu *et al.*, 2011) and the Hpa1_10–42_ coding sequence (Chen *et al.*, 2008*a*; Li *et al.*, 2013*a*) resulted in the generation of transgenic Y16:Hpa1_10–42_ plants. Six Y16:Hpa1_10–42_ lines (#1–#6) were characterized recently (Li *et al.*, 2013*b*) and they were further tested in this study. On the basis of *MYB44* responsiveness to aphids (Liu *et al.*, 2010), *44P*_*2000*_ truncated from the *MYB44* promoter (Liu *et al.*, 2011) was produced to direct the expression of *Hpa1*_*10–42*_ in transgenic wheat plants under attack by aphids. This hypothesis was validated as *Hpa1*_*10–42*_ was found to be expressed in Y16:Hpa1_10–42_ lines #1–#6 only when they were colonized with English grain aphid (Fig. 1). In contrast, no expression was detected in the parent Y16 plant irrespective of aphid infestations. Based on statistical analysis by one-tailed ANOVA and LSD test, the level of aphid-induced *Hpa1*_*10–42*_ expression is significantly (*P*<0.01) greater in Y16:Hpa1_10–42_#6 than in any of the other transgenic lines (Fig. 1).

### Hpa1_10–42_ expression in wheat represses the performance of English grain aphid

**Fig. 2.**
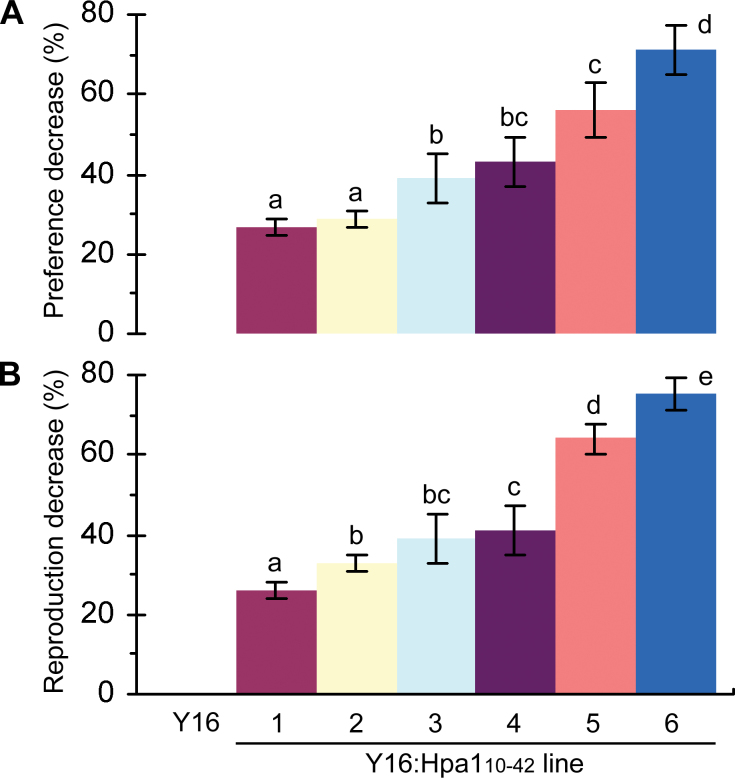
The effects of Hpa1_10–42_ expression on English grain aphid colonization and reproduction on wheat leaves. (A, B) Uniform 10-day-old adults of apterous and agamic aphid females were placed on the upper sides of the top two expanded leaves (10 aphids/leaf) of 30-day-old plants. Leaf colonies were surveyed 24h later. A total of 1200 aphids were monitored in four experimental repetitions (each containing 30 plants). The numerical values are means ±SDs, and different letters on the SD bars indicate significant differences by one-tailed ANOVA and LSD test (*P*<0.01). (A) Values of plant colonization preference were scored as the number of aphids that stayed in their colonies on leaves. The percentage decrease in the value of preference for a Y16:Hpa1_10–42_ line was calculated in comparison with the value of preference for Y16. (B) The reproduction rate is given as the ratio between the total number of newborn nymphs and the total number of adults on leaf colonies. The percentage decrease in the rate of reproduction on leaves of a Y16:Hpa1_10–42_ line was calculated in comparison with the rate of reproduction on Y16 leaves. (This figure is available in colour at *JXB* online.)

To correlate the responsiveness of *Hpa1*_*10–42*_ to English grain aphid with the insect performance on wheat plants, a large-scale population of the aphid was artificially placed on leaves of Y16 and Y16:Hpa1_10–42_ plants and the 24h fluctuation in leaf colonies was surveyed. A total of 1200 uniform individuals of apterous and agamic aphid females were monitored in four repetitions of the experiments. The number of aphids that stayed in their colonies on leaves was counted or the number of aphids that ran away from the leaf colonies was calculated over 24h. Colonization preference for a wheat genotype (Y16 or a Y16:Hpa1_10–42_ line) was indicated by the number of aphids in the leaf colony. If the value of colonization preference was decreased in a Y16:Hpa1_10–42_ line compared with Y16, this transgenic line was presumed to be more resistant than Y16 to aphid colonization. According to this criterion, resistance to aphid colonization is enhanced by 23–71% in Y16:Hpa1_10–42_#1–#6 relative to Y16 (Fig. 2A).

Aphid reproduction was assessed according to the value of the reproduction rate, quantified as the ratio between total numbers of nymphs produced in 5 d and total numbers of aphid adults that stayed in their original leaf colonies during the same period. A Y16:Hpa1_10–42_ line was presumed to be inhibitive to aphid reproduction if the reproduction rate was lower on the transgenic line compared with Y16. According to this criterion, all Y16:Hpa1_10–42_ lines are inhibitive to aphid reproduction and Y16:Hpa1_10–42_#1 is the most inhibitive (Fig. 2B).

Based on ANOVA and LSD test, Y16:Hpa1_10–42_ lines are significantly (*P*<0.01) different from Y16 in repressing the performance of English grain aphid (Fig. 2A, B). This analysis offers statistical evidence that transgenic expression of *Hpa1*_*10–42*_ in wheat induces resistance, effectively repressing both colonization and reproduction of aphids on the plant. Resistance levels are lower in Y16:Hpa1_10–42_#1 or #2 and moderate in #3–#5 in comparison with the highest level in #6 (Fig. 2A, B).

### Hpa1_10–42_ expression in wheat induces repression of the phloem-feeding behaviour of English grain aphid

To correlate colonization and reproduction performances with feeding behaviour of English grain aphid, the aphid feeding activities were studied by the EPG technique applied separately to 40 aphids that colonized leaves of Y16 and Y16:Hpa1_10–42_ plants. Feeding activities were depicted as different waveform patterns recognized according to the standard previously established (Tjallingii, 1988; Tjallingii and Esch, 1993) and widely used (Tjallingii, 2006; Will and van Bel, 2008; De Vos and Jander, 2009; C. Zhang *et al.*, 2011). Based on the EPG patterns, all the 40 aphids tested in five repetitions of the experiments for Y16 or a Y16:Hpa1_10–42_ line accomplished major steps of the feeding process, but aphid activities varied greatly depending on feeding stages.

**Fig. 3.**
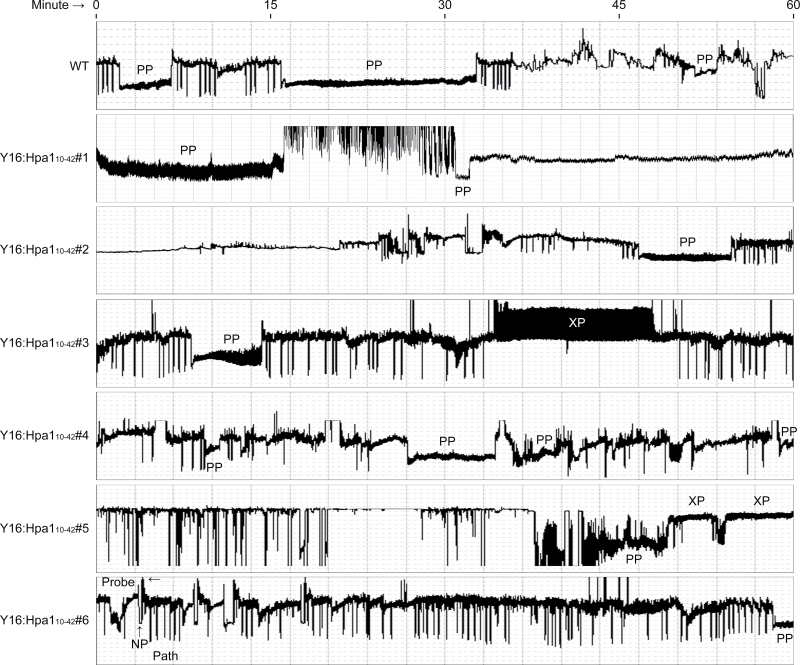
Electrical penetration graph (EPG) showing aphid feeding on leaves of the wheat cultivar Y16 and transgenic Y16:Hpa1_10–42_ lines. Uniform nymphs of the second instar were placed on the upper sides of the top first expanded leaves. The second hour parts of 6h EPG records are shown. Aphid feeding activities are divided into several distinct phases detected as distinct EPG waveforms. ‘Probe’ refers to aphid stylet puncturing of the plant cell; ‘NP’ indicates non-puncturing; ‘Path’ means pathways of stylet movements in fascicular cells; ‘XP’ and ‘PP’ refer to xylem and phloem phases when stylets take up soaps from the xylem and phloem, respectively. Note that other waveforms appear in some of the predominant PP spans. (This figure is available in colour at *JXB* online.)

Aphid feeding activities are divided into several distinct phases (C. Zhang *et al.*, 2011). Figure 3 shows those phases as waveform patterns or an EPG record span that contains a predominant waveform pattern. The non-puncturing phase (NP) indicates the stylet staying outside the cuticle. Cell puncturing (Probe) leads to the pathway phase (Path) in which the stylet penetrates between cells en route to the vascular tissue (Tjallingii and Esch, 1993; C. Zhang *et al.*, 2011). When the phloem of a wheat genotype is not a favourite source for feeding, the xylem phase (XP) may be observed while aphids try to suck soap from the xylem (C. Zhang *et al.*, 2011).

**Fig. 4.**
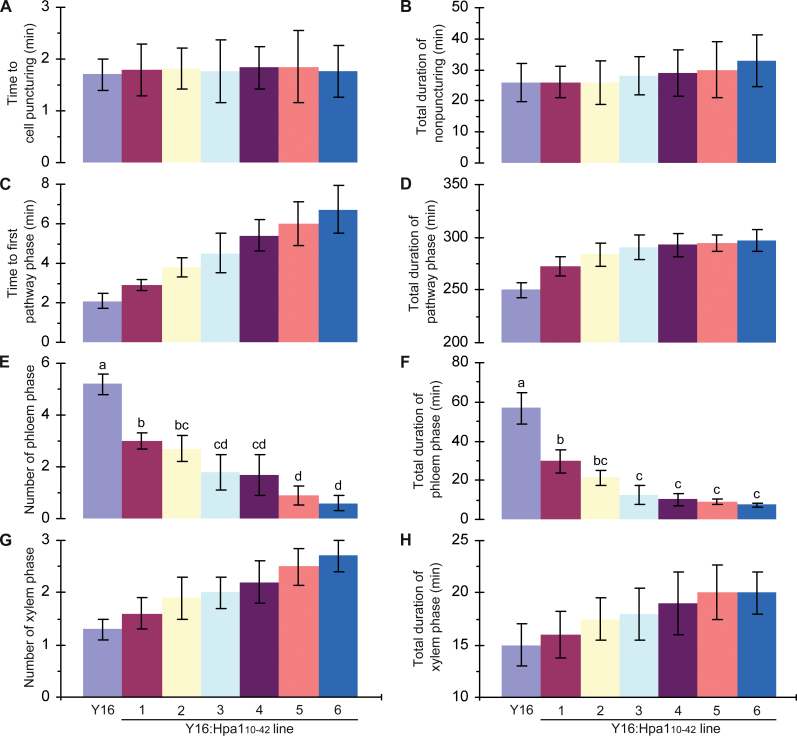
Quantitative presentation of 6h EPG records. Major parameters that reflect aphid feeding activities are provided in (A–H). Values shown are means ±SD of results obtained from monitoring of 40 aphids placed on the top first expanded leaves of five plants. In (E, F), different letters indicate significant differences among compared plants by one-tailed ANOVA and LSD test (*P*<0.01). (This figure is available in colour at *JXB* online.)

Figure 4 shows 6h EPG analyses of aphid feeding from leaves of Y16 and Y16:Hpa1_10–42_ plants. In the 6h EPG record, the time to the first cell puncturing (Fig. 4A) and the total duration of the non-puncturing phase (Fig. 4B) were similar in all plants. Time to the first pathway phase (Fig. 4C) and duration of this phase (Fig. 4D) were longer in Y16:Hpa1_10–42_ lines than in the Y16 plant. The pathway phase represents an insect’s efforts in navigating the phloem and preparing to ingest sap from sieve elements (Tjallingii, 2006; C. Zhang *et al.*, 2011). It was evident that the aphid activities outside leaf cells had no obvious changes (Fig. 4A, B), whereas aphids took much longer time in the pathway phase when they were feeding from Y16:Hpa1_10–42_ plants than from Y16 plants (Fig. 4C, D). Clearly, the expression of *Hpa1*_*10–42*_ in transgenic wheat lines impeded the feeding activities of aphids once their stylets penetrate the leaf cells.

Subsequent to the pathway phase, aphids may proceed to the phloem phase (Fig. 3) in which ingestion of the phloem sap may occur (C. Zhang *et al.*, 2011). Aphid feeding activities in the phloem phase were significantly (ANOVA and LSD, *P*<0.01) repressed in Y16:Hpa1_10–42_ lines compared with the Y16 plant. In Y16:Hpa1_10–42_ lines, the number in the phloem phase was small (Fig. 4E) while the total duration of this phase was much shorter (Fig. 4F). In contrast, the number in the xylem phase was greater and the total duration of this phase was longer on leaves of Y16:Hpa1_10–42_ compared with Y16 (Fig. 4G, H), indicating that the Y16:Hpa1_10–42_ phloem was not a favourite source for feeding.

Statistical analysis by ANOVA and LSD (*P*<0.01) confirmed differences between Y16:Hpa1_10–42_ and Y16 plants in the number in the phloem phase and total duration of this phase in a 6h EPG record. In particular, decreases were significant in both the number in the phloem phase and the duration of this phase when aphids were feeding on Y16:Hpa1_10–42_ lines in contrast to the Y16 plant. This analysis suggests that aphid feeding from the phloem is repressed due to the expression of *Hpa1*_*10–42*_ in transgenic wheat lines. Also, of the six transgenic lines, Y16:Hpa_10–42_#6 is most inhibitive to phloem feeding (Fig. 4E, F).

### Hpa1_10–42_ expression in wheat induces the phloem-based defence

**Fig. 5.**
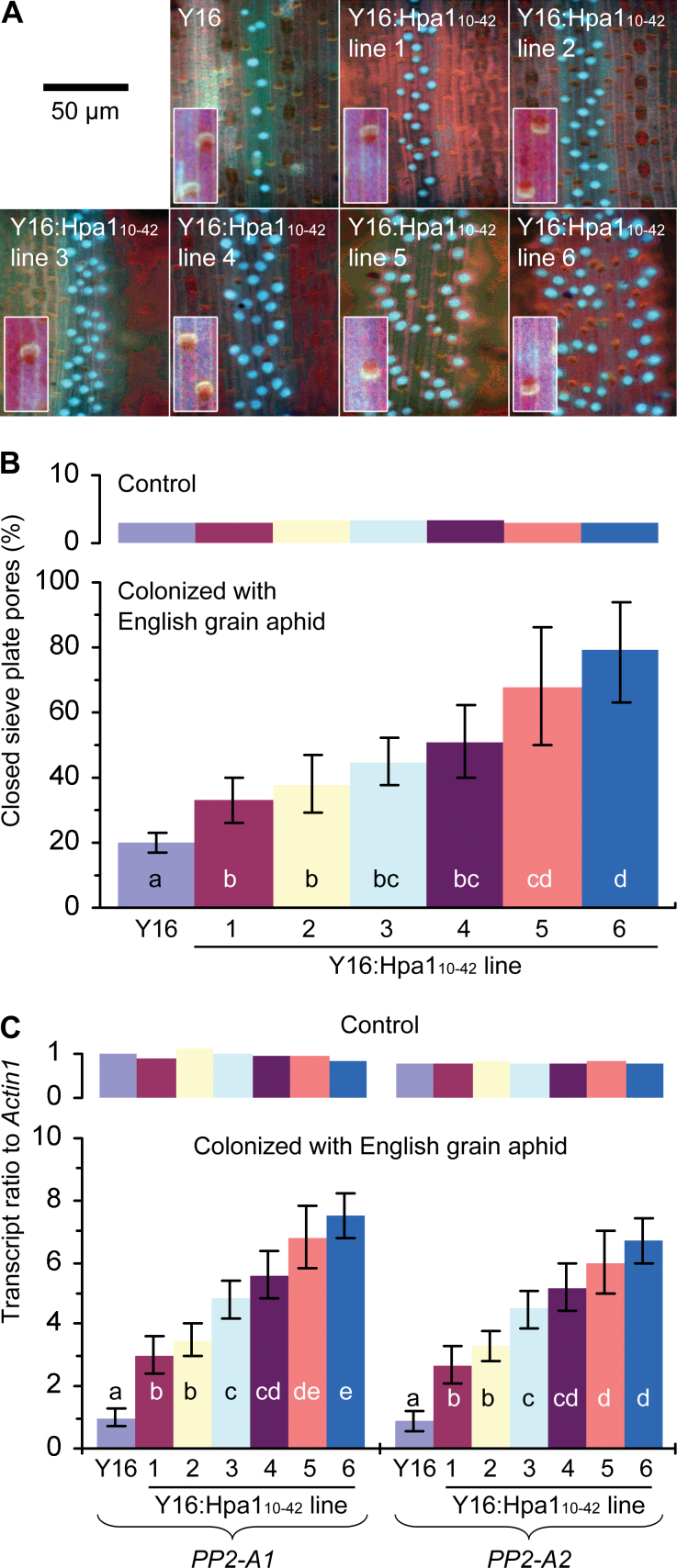
Callose deposition and *PP2-A* expression in leaves of Y16 and Y16:Hpa1_10–42_ plants. (A–C) Plants were colonized with English grain aphid or not colonized in the control. Six hours later, callose deposition and *PP2-A* expression were analysed. (A) In plants colonized with aphids, callose deposition in the vascular bundles of leaf middle veins was visualized as a blue colour by staining the leaves with aniline blue. Insets show sieve plates from leaves of control plants. (B) Proportions of callose-closed sieve plate pores were scored from imaging data equivalent to those in (A). In total 750–1250 sieve plates were observed in three experimental repeats for a genotype of plant (Y16 or each of the Y16:Hpa1_10–42_ lines). Data shown are mean values ±SD. (C) *PP2-A*/*Actin1* transcript ratios were quantified by real-time RT–PCR as mean values ±SD of results from three experimental repeats (15 plants/repeat). In (B, C), different letters on the bar graphs indicate significant differences among compared plants by one-tailed ANOVA and LSD test (*P*<0.01).

**Fig. 6.**
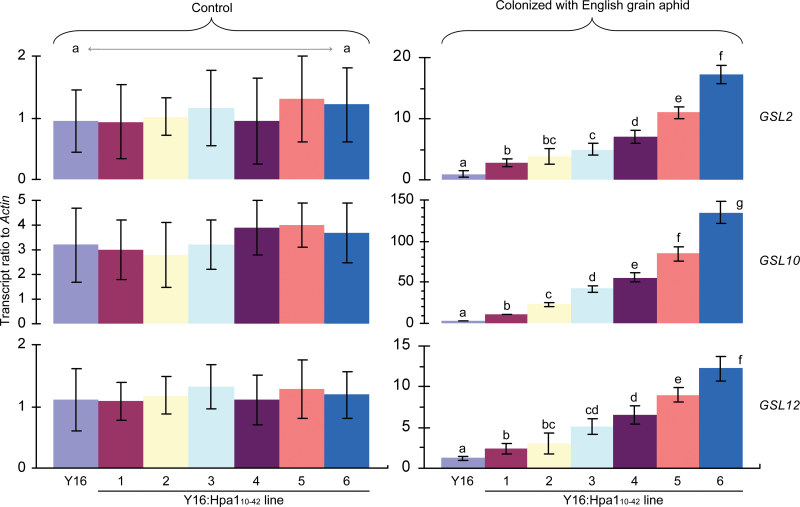
The expression of *GSL* genes in leaves of Y16 and Y16:Hpa1_10–42_ plants. Plants were colonized with English grain aphid or not colonized in the control, and gene expression was analysed 6h later. Data shown are mean values ±SD of results from three experimental repeats (15 plants per repeat). In the left vertical panels, different letters on the bar graphs indicate significant differences among compared plants by one-tailed ANOVA and LSD test (*P*<0.01). (This figure is available in colour at *JXB* online.)

To correlate the repression of aphid feeding from the phloem with the phloem-based defence, callose deposition and the expression of *PP2A* and *GSL* genes in leaves were analysed to reveal if the defence might differ in Y16:Hpa1_10–42_ lines from that in the Y16 plant under attack by English grain aphid. As shown in Fig. 5A, callose deposition was detected predominantly in vascular bundles located in the middle veins of leaves and the amounts deposited are more substantial in leaves of Y16:Hpa1_10–42_ than in those of the parent plant. Callose was found to be predominantly deposited on sieve plates to close sieve plate pores. The proportions of closed sieve plate pores were significantly (*P*<0.01 by ANOVA and LSD) greater in Y16:Hpa1_10–42_ lines than in the Y16 plant (Fig. 5B). Thus, callose deposition and closure of sieve plate pores by the deposit were enhanced in Y16:Hpa1_10–42_ lines in contrast to the Y16 plant. Similarly, the expression of *PP2-A1* and *PP2-A2* was significantly (*P*<0.01 by ANOVA and LSD) enhanced in Y16:Hpa1_10–42_ lines compared with the Y16 plant (Fig. 5C). In Y16:Hpa1_10–42_ plants, moreover, significant (*P*<0.01 by ANOVA and LSD) enhancements were also found in the expression of three of nine *GSL* genes identified in the wheat genome (Fig. 6; Supplementary Figs S1 and S2 at *JXB* online). The three genes were *GSL2*, *GSL10*, and *GSL12*, enhanced in expression levels accordingly by 3–19, 4–45, and 2–10 times in Y16:Hpa1_10–42_ lines compared with in Y16 (Fig. 6). Clearly, the phloem-based defence, shown as the closure of sieve plate pores by callose deposits and the expression of *PP2-A*, *GSL2*, *GSL10*, and *GSL12* genes, is activated due to the expression of *Hpa1*_*10–42*_ in transgenic wheat lines, especially Y16:Hpa1_10–42_#6 (Figs 5A–C, 6).

When plants were not colonized with aphids, *PP2-A*, *GSL2*, *GSL10*, and *GSL12* transcripts were detected in leaves at steady-state levels (equivalent in Y16 and Y16:Hpa1_10–42_#6; Figs 5C, 6) while callose was not found to be substantially deposited at sieve plates (Fig. 5A, B). Therefore, the phloem-based defence is similar to the Hpa1_10–42_ expression (Fig. 1B) in terms of the requirement for induction. Indeed, the phloem-based defence is an induced trait and does not develop without induction by aphid infestations under the conditions of this study (Figs 5, 6).

### Hpa1_10–42_-induced phloem-based defence is regulated by ethylene signalling

Two independent experiments were performed on the Y16 plant and the transgenic line Y16:Hpa1_10–42_#6 to address whether the ethylene signalling pathway plays a role in Hpa1_10–42_-induced phloem-based defence of wheat. Y16:Hpa1_10–42_#6 was used in the experiments because it acquires the greatest extent of phloem-based defence of the six Y16:Hpa1_10–42_ lines already tested (Figs 5, 6).

**Fig. 7.**
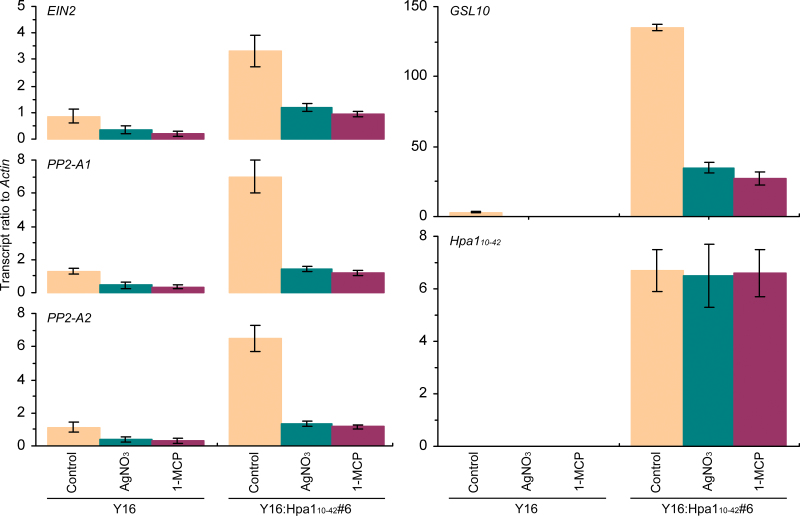
The effects of ethylene signalling inhibitors on the expression of *EIN2*, *PP2-A*, and *GSL* genes tested in comparison with *Hpa1*_*10–42*_. Plants were colonized with aphids and simultaneously treated with water (control) and with the ethylene signalling inhibitor AgNO_3_ or 1-methylcyclopropene (1-MCP). Six hours later, gene expression was analysed. Data shown are mean values ±SD of results from three experimental repeats (10 plants per repeat). (This figure is available in colour at *JXB* online.)

**Fig. 8.**
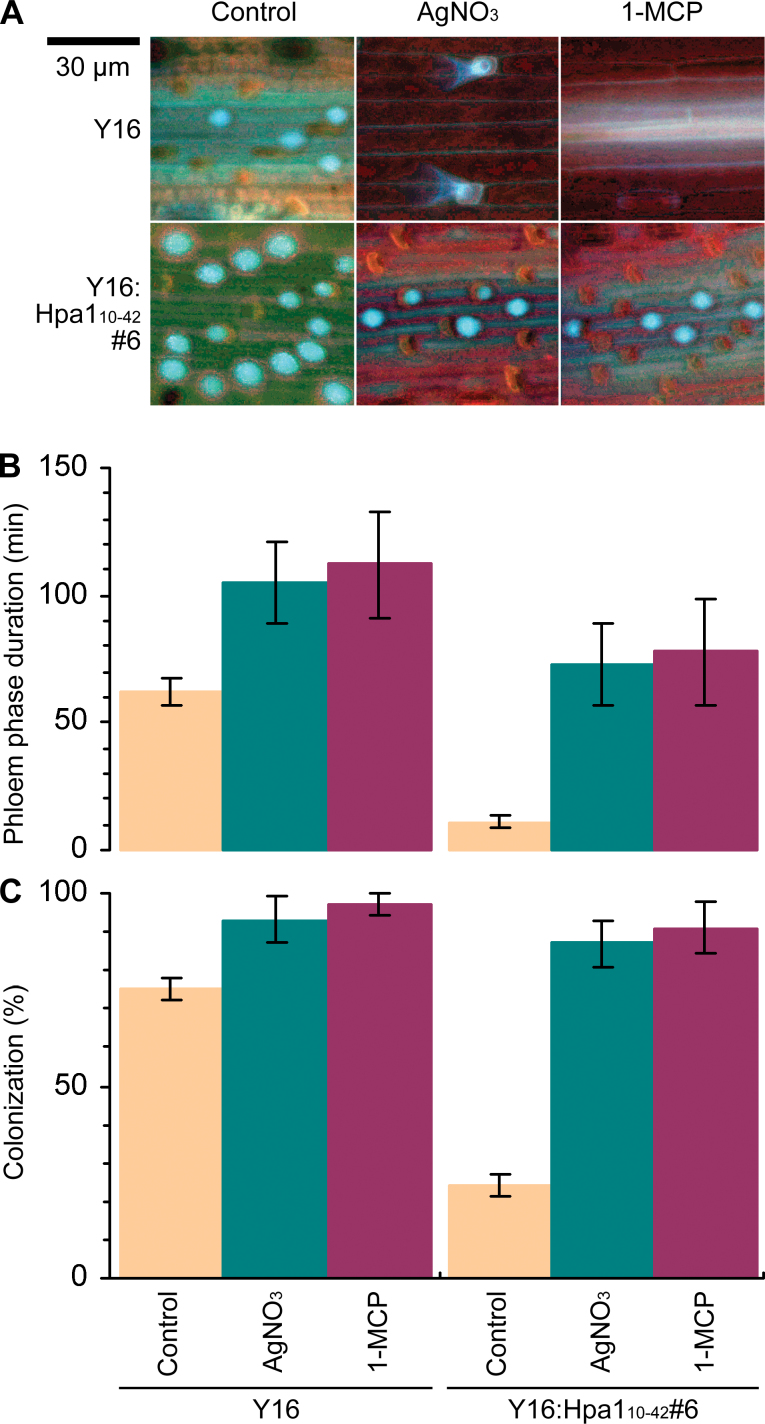
The effects of ethylene signalling inhibitors on leaf callose deposition and aphid performance on the plant. (A–C) Plants were colonized with aphids and simultaneously treated with water (control), AgNO_3_, or 1-MCP. (A) Six hours later, callose deposition was detected. (B, C) A further 18h later, the phloem feeding duration was scored by EPG, and the proportions of aphids which stayed in leaf colonies were calculated. Data shown are mean values ±SD of results from three experimental repeats.

The first experiment was devised to determine expression of the *EIN2*, *PP2-A*, and *GSL* genes in Y16 and Y16:Hpa1_10–42_#6 plants colonized with English grain aphid. In this case, *EIN2* was expressed in coordination with *PP2-A2*, *PP2-A2*, *GSL2*, *GSL10*, and *GSL12*, and their expression levels were highly elevated in Y16:Hpa1_10–42_#6 compared with the steady-state level of gene expression in Y16 (Fig. 7; Supplementary Fig. S3 at *JXB* online). Callose deposition on sieve plates was also enhanced in Y16:Hpa1_10–42_ (Fig. 8A). Thus, *EIN2* expression is coordinated with the phloem-based defence response, indicating that ethylene signalling may function through *EIN2* to regulate Hpa1_10–42_-induced phloem-based defence.

This hypothesis was tested in a second experiment, in which Y16 and Y16:Hpa1_10–42_#6 plants were treated with the ethylene signalling inhibitor AgNO_3_ (Dong *et al.*, 2004) or 1-MCP (Zhang *et al.*, 2007; Ren *et al.*, 2008) and the subsequent effects on the phloem-based defence were analysed. As shown in Fig. 7, a marked proportion of Hpa1_10–42_-enhanced *EIN2* expression was eliminated by treating Y16:Hpa1_10–42_ plants with 1-MCP or AgNO_3_. The pharmacological treatment further had inhibitory effects on Hpa1_10–42_-induced enhancements in *PP2-A* and *GSL* expression (Fig. 7) and on the closure of sieve plate pores by callose deposits (Fig. 8A). Thus, the inhibition of ethylene signalling indeed impaired the phloem-based defence. This defect in Y16:Hpa1_10–42_#6 further impaired resistance to aphids, or, inversely, was favouring the phloem-feeding behaviour of aphids (Fig. 8B) and their performance in colonizing the plant (Fig. 8C). In the Y16 plant, the pharmacological treatment also caused inhibitory effects on *EIN2* expression and the phloem-based defence (Figs 7, 8A), increasing the abilities of aphids to establish colonies and complete reproduction on the plant (Fig. 8C, D). In Y16:Hpa1_10–42_, however, neither AgNO_3_ nor 1-MCP caused an inhibitory effect on Hpa1_10–42_ expression, in contrast to the inhibition on *EIN2* (Fig. 7), suggesting that both inhibitors executed their inhibitory role by blocking ethylene signalling for *EIN2* expression, rather than directly affecting the role of Hpa1_10–42_ in inducing the phloem-based defence. Taken together, data obtained from these two independent experiments support the idea that Hpa1_10–42_-induced phloem-based defence is subject to ethylene signalling in wheat under attack by English grain aphid.

### Hpa1_10–42_ expression enhances the growth of aerial parts of wheat but represses root development

**Fig. 9.**
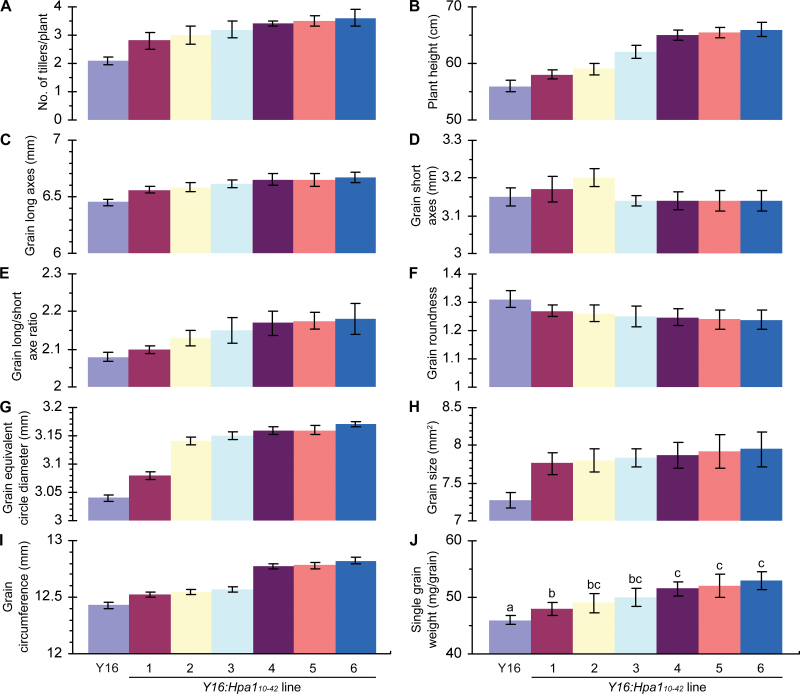
Analyses of wheat growth and grain characters. (A, B) Tillers were counted after the first flowering day and plant height was measured based on the tallest ear. (C–J) Morphological characters of grains were analysed by a seed analyser. (A–J) Data shown are mean values ±SDs of results from three experimental repeats (50 plants or 15g of grains per repeat). In (J), different letters on the SD bars indicate significant differences among compared plants by one-tailed ANOVA and LSD test (*P*<0.01). (This figure is available in colour at *JXB* online.)

**Fig. 10.**
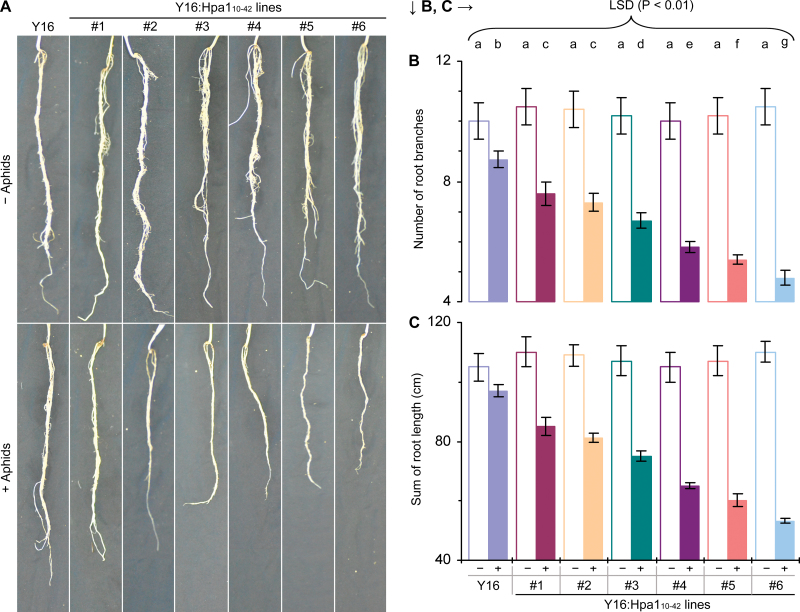
Observations of wheat root systems. (A) Roots from 25-day-old plants grown in pots. Plants were protected from aphid infestations (– Aphids) or leaves of 10-day-old plants were colonized with aphid nymphs (+ Aphids). (B, C) Quantification of root growth and branching of 25-day-old plants. The symbol ‘–’ indicates the absence of colonization with aphids, and ‘+’ indicates leaf colonization with aphid nymphs as in (A). Data shown are mean values ±SDs of results from three experimental repeats (15 plants per repeat). Different letters on the SD graphs indicate significant differences (*P*<0.01). (This figure is available in colour at *JXB* online.)

To assess the effects of Hpa1_10–42_ on agronomic traits of wheat, six Y16:Hpa1_10–42_ lines were compared with the Y16 plant in terms of vegetative growth and grain production in a glass-equipped greenhouse. As Hpa1_10–42_ expression needs induction, plants were colonized with a small amount of English grain aphid nymphs. The artificial colonization was performed three times at the 10-day-old seedling, littering, and flowering stages, respectively. Under this condition, all Y16:Hpa1_10–42_ lines produced more tillers (Fig. 9A) and had a greater plant height than Y16, while Y16:Hpa1_10–42_#6 acquired the most vigorous growth (Fig. 9B). Interestingly, the root development seemed different in Y16 and Y16:Hpa1_10–42_ plants depending on whether or not plant leaves were colonized with aphids. In the absence of aphid colonization, Y16:Hpa1_10–42_ lines apparently resembled the Y16 plant in terms of root development (Fig. 10A). After growth for 25 d in soil (Fig. 10) or in the nutrient solution (Supplementary Fig. S4 at *JXB* online) under insect-free conditions, all plants were similar in the number of root branches (Fig. 10B) and in the total length of root branches in total (Fig. 10C). If leaves of 10-day-old plants were colonized with aphids, root branching and growth in the subsequent 15 d were remarkably repressed in Y16 and Y16:Hpa1_10–42_ plants. However, the extents by which the aphid colonization repressed root branching and growth were significantly (*P*<0.01) higher in Y16:Hpa1_10–42_ lines than in the Y16 plant (Fig. 10B, C).

### Hpa1_10–42_ expression increases grain yield of wheat in the presence of a small amount of aphid infestation

Morphological characters of grains were analysed in detail. Morphological characters of grains are often used to assess grain quality, and, if grains of two wheat cultivars are analysed, high quality is indicated by greater values of grain roundness and equivalent circle diameter but a smaller value of the long to short axis ratio (Shouche *et al.*, 2001). Based on this evaluation criterion, grains of Y16:Hpa1_10–42_ lines do not conform to all parameters of high quality (Fig. 9C–F). However, both the grain size and single grain weight of Y16:Hpa1_10–42_ lines are greater than those of the parent (Fig. 9G–J). Therefore, the beneficial effects of Hpa1_10–42_ expression on agronomic characters of wheat are to enhance the vegetative growth and increase grain yield even if the Y16:Hpa1_10–42_ grains do not show high quality in all the morphological parameters. Of the six transgenic lines, moreover, Y16:Hpa1_10–42_#6 acquires the greatest growth enhancement and grain yield increase (ANOVA and LSD, *P*<0.01). In addition, major characters of grains are similar in Y16 and Y16:Hpa1_10–42_#6 plants if they are not colonized with aphids (Supplementary Fig. S4 and Table S2 at *JXB* online).

## Discussion

On the basis of previous demonstrations of the defensive and/or developmental roles of harpin proteins expressed as full-length copies in transgenic plants (Peng *et al.*, 2004; Miao *et al.*, 2010*a*, *b*; L. Zhang *et al.*, 2011; Sang *et al.*, 2012), this study is focused on the defensive role of Hpa1_10–42_ as a robust functional fragment, isolated from the Hpa1 protein sequence (Wu *et al.*, 2007; Chen *et al.*, 2008*a*, *b*) and expressed in an agriculturally significant crop. Following characterizations of Hpa1_10–42_ in regard to its physiological, developmental, and pathological roles (Wu *et al.*, 2007; Chen *et al.*, 2008*a*), this study analyses a novel function that the transgenic expression of Hpa1_10–42_ performs in wheat phloem-based defence against the English grain aphid. This novel function and associated regulatory components have been elucidated with several sets of evidence summarized below.

First, the aphid infestation induces substantial expression of Hpa1_10–42_ under the direction of the *44P*_*2000*_ promoter in transgenic wheat lines (Fig. 1), confirming that *44P*_*2000*_ is responsive to insect attacks in addition to harpin or ethylene (Liu *et al.*, 2011; Lü *et al.*, 2013). So far, three species of insects, the green peach aphid (Lü *et al.*, 2013), the English grain aphid (this study), and the diamondback moth (*Plutella xylostella* L.) (Lü *et al.*, 2013), have been shown to induce *44P*_*2000*_-directed Hpa1_10–42_ expression. Due to the induced activity of *44P*_*2000*_, Hpa1_10–42_ expression in transgenic wheat lines is an induced but not a constitutive trait and is not likely to cause subsequent effects on the phloem-based defence in the plant without induction by aphid infestation, for instance. This provides a basis for the genetic engineering design for ‘insect-induced resistance to insects’ (Lü *et al.*, 2013).

Secondly, the Hpa1_10–42_ expression causes a repression in the performance of English grain aphid (Fig. 2) in correlation with a repression of phloem-feeding activities of the insect on wheat (Figs 3, 4). In a previous study, the design for ‘insect-induced resistance to insects’ was tested by observing the inhibitory effect of a primary infestation on a secondary infestation of insects on *Arabidopsis* (Lü *et al.*, 2013). In this case, primary infestation of the green peach aphid nymphs or diamondback month caterpillars induces resistance to secondary infestations of both insects. The present study shows that Hpa1_10–42_-induced resistance is effective in repressing the performance and behaviour of English grain aphid in the concurrent infestation.

Thirdly, Hpa1_10–42_-induced phloem-based defence observed in transgenic wheat lines that were colonized with English grain aphid involves enhanced expression of defence-associated genes (*PP2-A*, *GSL2*, *GSL10*, and *GSL12*) and the closure of sieve plate pores by callose deposition under regulation by ethylene signalling (Figs 5–8; Supplementary Figs S1–S3 at *JXB* online). At present, however, it is not known whether *PP2-A1* and *PP2-A2* or the three *GSL* genes have functional redundancy. It is also not known whether *GSL5* affects the phloem-based defence in wheat as in *Arabidopsis* (Lü *et al.*, 2011) since the *GSL5* orthologue has not been identified in wheat (Voigt *et al.*, 2006; Burton *et al.*, 2008; Taketa *et al.*, 2012).

The role of ethylene signalling in Hpa1_10–42_-induced phloem-based defence offers additional evidence to previous demonstrations that the induction of plant defence responses through activating phytohormone signalling pathways is a conserved function of harpin proteins in a variety of plant species (Dong *et al.*, 1999, 2004, 2005; Kim and Beer, 2000; Peng *et al.*, 2003, 2004; Liu *et al.*, 2006; Chen *et al.*, 2008*a*, *b*; Liu *et al.*, 2011; Lü *et al.*, 2011, 2013; C. Zhang *et al.*, 2011). In this regard, one important facet of this study is to extend the defensive scope of plant engineering with a harpin protein, from disease resistance (Dong *et al.*, 1999, 2004; Chen *et al.*, 2008*a*, *b*) and drought tolerance (Dong *et al.*, 2004; Zhang *et al.*, 2007) to resistance against insect pests, and to extend the defensive roles from biological model plants such as *Arabidopsis* (Dong *et al.*, 2005; Lü *et al.*, 2013) to agriculturally significant crops such as wheat. In particular, coincident roles of Hpa1_10–42_ in inducing the phloem-based defence and altering agronomic traits, especially enhancing vegetative growth and increasing grain output (Fig. 9), suggest that the defensive and developmental roles of Hpa1_10–42_ can be integrated into breeding germplasm of the agriculturally significant crop.

However, Hpa1_10–42_ may cause fitness consequences in transgenic wheat lines, such as repression of root branching and growth observed in this study (Fig. 10). The repressive effect may be attributed to an elevated level of ethylene based on previous demonstrations that the external application of a harpin protein induces the production of ethylene in aerial parts (Dong *et al.*, 2004; Zhang *et al.*, 2007; Ren *et al.*, 2008) and roots (Dong *et al.*, 2004) of *Arabidopsis*, and that the application of ethylene to wheat inhibits plant root elongation (Huang *et al.*, 1997). The repressive effect of Hpa1_10–42_ on root development is likely to impair the agricultural value of transgenic wheat lines in planting areas where drought is a constant challenge.

This notion is of practical significance in regard to the simultaneous improvement of developmental and defensive traits by integrating the development–defence cross-talk mechanism into breeding germplasm of crops. Plants utilize sophisticated strategies to regulate the cross-talk and thereby minimize developmental cost and fitness consequences of defence responses to attacks by pathogens or insect pests (Dangl *et al.*, 1996; Yu *et al.*, 1998; Chen *et al.*, 2008*a*, *b*; Mukhtar *et al.*, 2009; Spoel *et al.*, 2009). One of the strategies is to inactivate defence signal transduction to reduce the fitness consequences that are associated with a constitutive defence response in the absence of a pathogen or insect attack (Mukhtar *et al.*, 2009; Spoel *et al.*, 2009). Alternatives could be provided by the functional mode of harpin proteins as they induce development and defence cross-talk in different plant species (Peng *et al.*, 2004; Wu *et al.*, 2007; Chen *et al.*, 2008*a*, *b*). In this regard, the demonstration of defensive and developmental roles of Hpa1_10–42_ expression in wheat represents a substantial step toward simultaneous improvements of defensive and agronomic traits by the genetic engineering technique. It is quite fascinating that a small amount of aphid infestation induces the developmental function of Hpa1_10–42_ in addition to its defensive role due to the use of the multifunctional promoter (Liu *et al.*, 2011; Lü *et al.*, 2013). Owing to the presence of such a promoter, the ‘insect-induced resistance to insects’ strategy has dual consequences, increasing the agronomic value of grain and enhancing the phloem-based defence against English grain aphid.

The phloem-based defence is a common defensive mechanism that all plants utilize to resist attacks by phloem-feeding herbivores (Kehr, 2006; Tjallingii, 2006; C. Zhang *et al.*, 2011). This mechanism has been shown to impede aphid infestations effectively in different plant species including wheat and other crops (Kehr, 2006; Tjallingii, 2006; Will and van Bel, 2006, 2008; Lü *et al.*, 2011, 2013; C. Zhang *et al.*, 2011). The broad significance and universal value of the defensive mechanism can also be found in phloem puncturing as a highly specialized and commonly utilized mode of feeding irrespective of the aphid species and the plants they attack (Tjallingii and Esch, 1993; Kehr, 2006; Tjallingii, 2006; Will and van Bel, 2006, 2008; Lü *et al.*, 2011; C. Zhang *et al.*, 2011). Therefore, it is likely that Hpa1_10–42_-induced phloem-based defence can be effective to resist other species of wheat aphids, such as *Schizaphis graminum* Rondani and *Rhopalosiphum padi* Linnaeus, in addition to *Sitobion avenae* Fabricius (English grain aphid). However, at least two additional conditions should be considered in regard to the potential of agricultural use of Hpa1_10–42_-expressing plants. First, it is necessary to study in the future whether the Hpa1_10–42_ expression is effective to resist simultaneous infestations of different species of wheat aphids. Secondly, many experiments are required to evaluate the environmental fitness of Hpa1_10–42_-expressing plants under natural field conditions.

## Supplementary data

Supplementary data are available at *JXB* online.

Figure S1. The expression of *GSL3*, *GSL6*, and *GSL8* in leaves of Y16 and Y16:Hpa1_10–42_ plants.

Figure S2. The expression of *GSL19*, *GSL22*, and *GSL23* in leaves of Y16 and Y16:Hpa1_10–42_ plants.

Figure S3. The effects of ethylene signalling inhibitors on the expression of *GSL2* and *GSL12* genes.

Figure S4. The effects of leaf colonization with aphids on the root growth of Y16 and Y16:Hpa1_10–42_ plants.

Table S1. Information on genes analysed and primers used in this study.

Table S2. Characters of seeds from plants that were not colonized with aphids.

Supplementary Data
