# Supplementary material for: Transgenic expression of a functional fragment of harpin protein Hpa1 in wheat induces the phloem-based defence against English grain aphid
Source: J Exp Bot. 2014 Mar 25;65(6):1439–53. doi: 10.1093/jxb/ert488 (PMC3967084; doi:10.1093/jxb/ert488)
Supplement: Supplementary Data [file supp_65_6_1439__index.html]

Transgenic expression of a functional fragment of harpin protein Hpa1 in wheat induces the phloem-based defence against English grain aphid — Supplementary Data 

# Transgenic expression of a functional fragment of harpin protein Hpa1 in wheat induces the phloem-based defence against English grain aphid

## Supplementary Data

Data files

**Files in this Data Supplement:**

- Supplementary Data - Supplementary Data
