# Supplementary material for: Transgenic expression of a functional fragment of harpin protein Hpa1 in wheat induces the phloem-based defence against English grain aphid
Source: J Exp Bot. 2014 Mar 25;65(6):1439–53. doi: 10.1093/jxb/ert488 (PMC3967084; doi:10.1093/jxb/ert488)
Supplement: Supplementary Data [file supp_ert488_jexbot103762_file001.pdf]

Supplementary Data

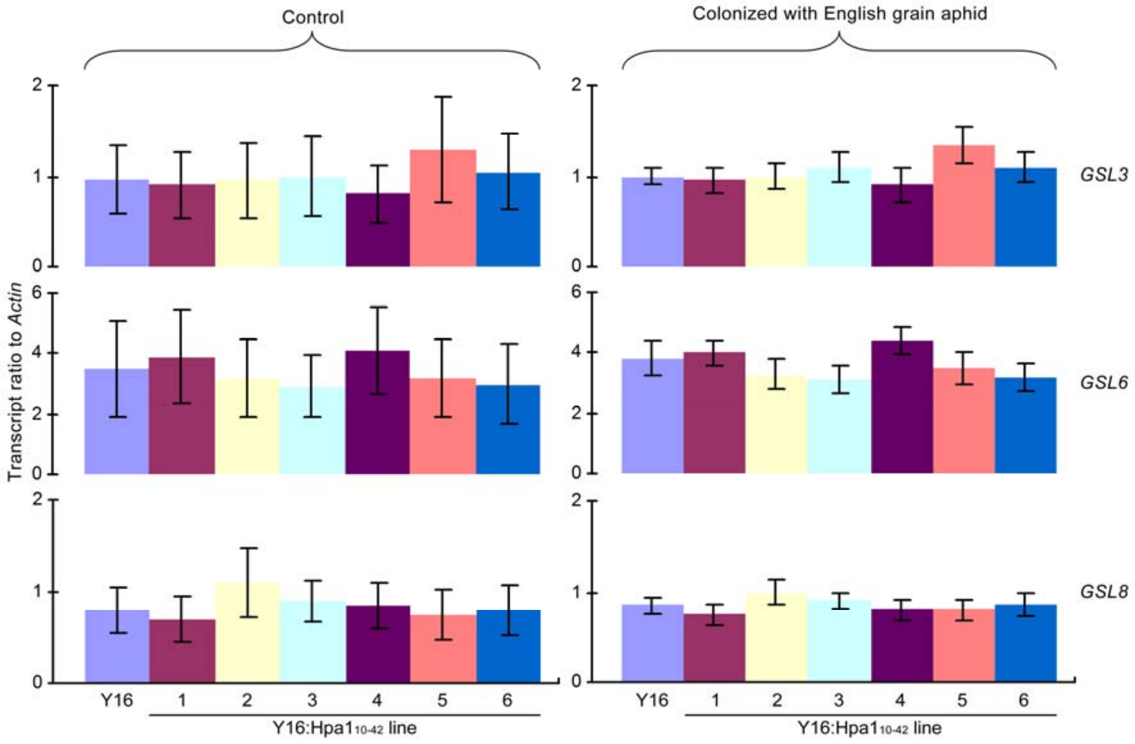

Fig. S1. The expression of *GSL3*, *GSL6*, and *GSL8* in leaves of Y16 and Y16:Hpa1$_{10\text{-}42}$ plants. Plants were colonized with English grain aphid or not colonized in control and gene expression was analyzed six hours later. Data shown are mean values ± SD bars of results from three experimental repeats (15 plants/repeat).

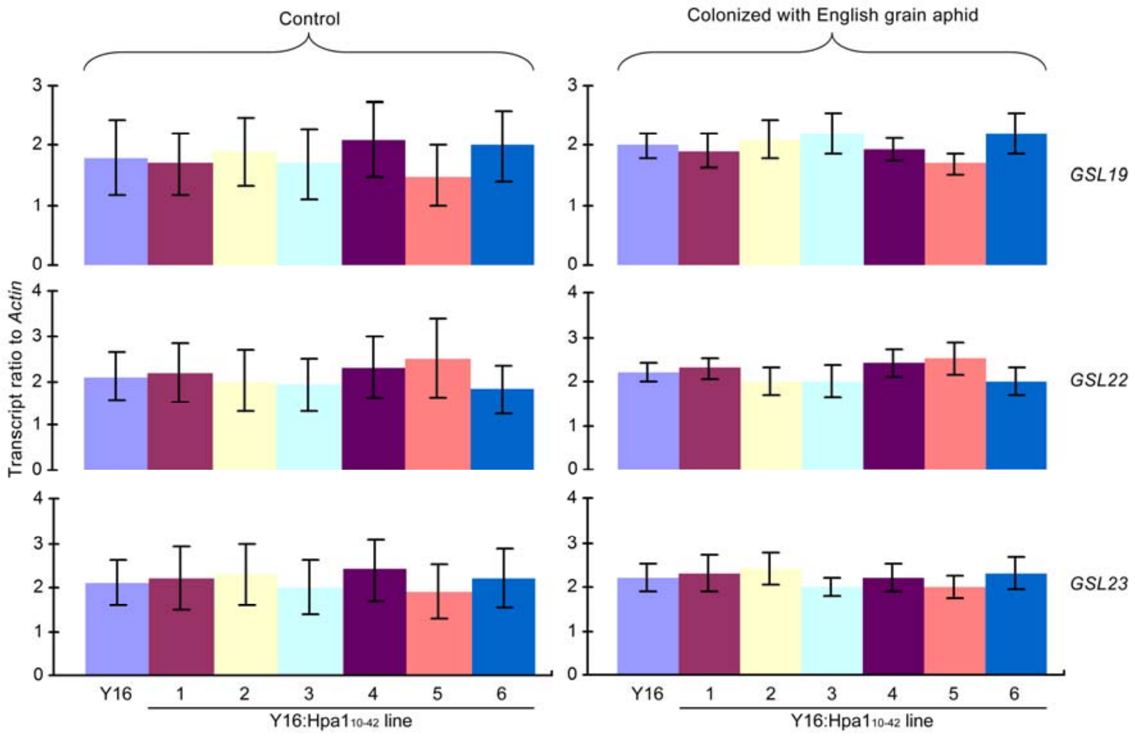

Fig. S2. The expression of *GSL19*, *GSL22*, and *GSL23* in leaves of Y16 and Y16:Hpa1$_{10\text{-}42}$ plants. Plants were colonized with English grain aphid or not colonized in control and gene expression was analyzed six hours later. Data shown are mean values ± SD bars of results from three experimental repeats (15 plants/repeat).

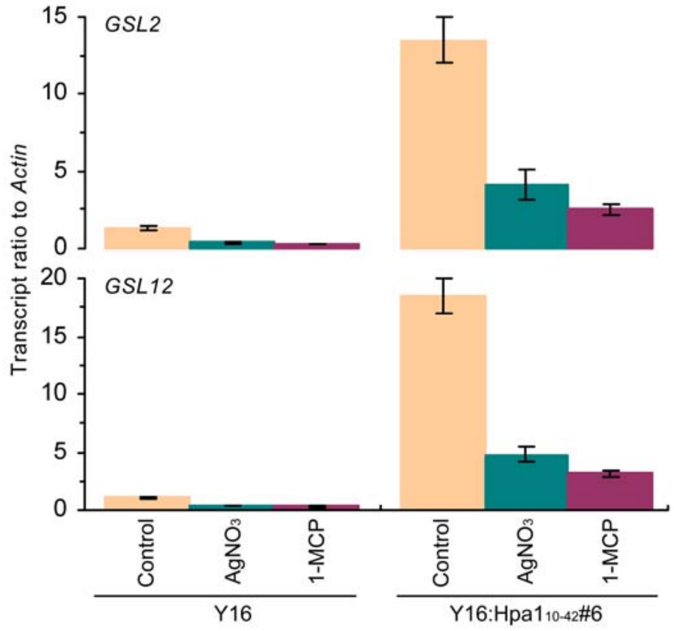

Fig. S3. The effects of ethylene signaling inhibitors on the expression of *GSL2* and *GSL12* genes. Plants were colonized with aphids and simultaneously treated with water (control), $AgNO_3$, or 1-MCP. Six hours later, gene expression was analyzed. Data shown are mean values ± SD bars of results from three experimental repeats (10 plants/repeat).

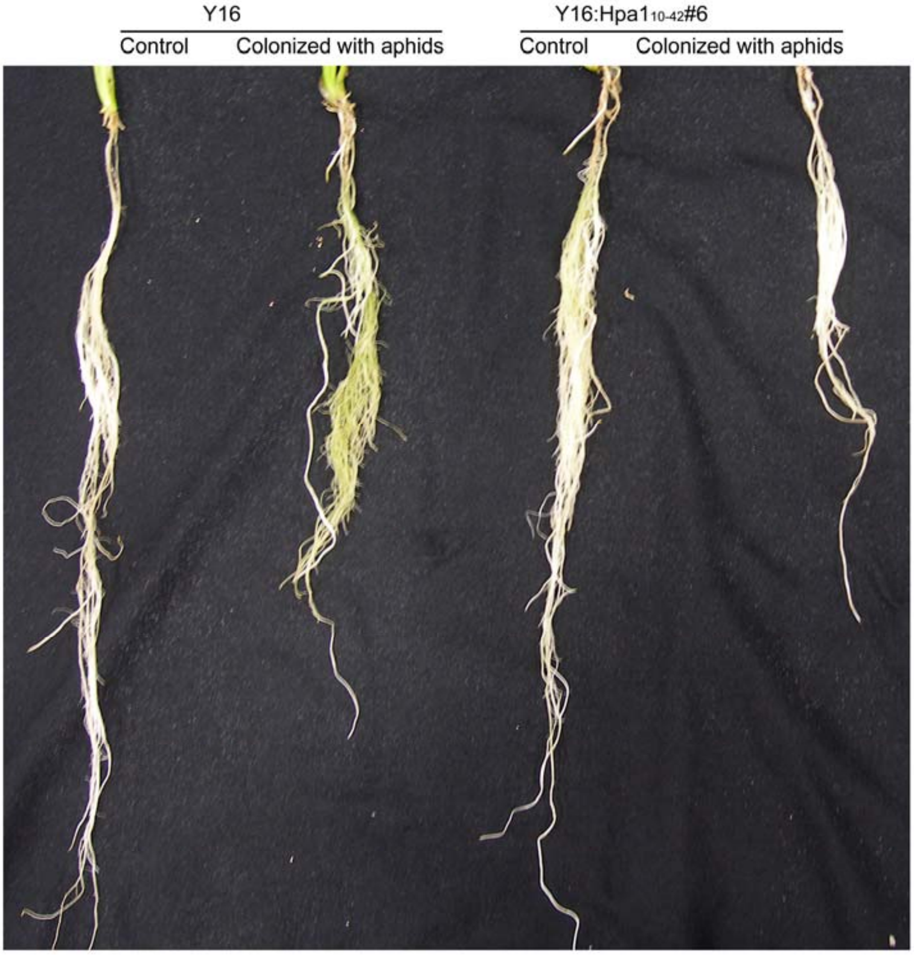

Fig. S4. The effects of leaf colonization with aphids on the root growth of Y16 and Y16:Hpa1$_{10\text{-}42}$ plants. Plants were cultured in the Hogland nutrition liquid and kept from aphid infestations in the experimental control group. In the aphid colonization group, the upper sides of the top two expanded leaves of 15-day–old plants were colonized with uniform 10-day–old aphids (5 aphids/leaf). Twenty days later, roots were excised and photographed. Each root photo represents 15 plants.

Table S1. Information on genes analyzed and primers used in this study

Gene	GenBank accession number	Primers	Region in the gene sequence	RT-PCR product size (bp)
Actin1	CA483746	5'-GGCCGGAGGCCGAGGGGG-3', 5'-CGGTGGCTTTGCCACAAA-3'	1–213	213
Hpa1$_{10\text{-}42}$	AY875714	5'-ATGGGCAGCGCGTCCAACTTC-3', 5'-TCAGTGGTGGTGGTGGTGGTGCA-3'	33–126	99
**PP2-A1*	NM_118104	5'-CACTTCGACCTCCTAGGCCG-3', 5'-CTCATACATTGAGAAAGTGAT-3'	514–739	199
**PP2-A2*	NM_001160782	5'-GTCCAGCTGCTACTGAAAAGGA-3', 5'-CTTTGCAACCTCTGATCTTAC-3'	108–312	205
GSL2	DQ0864383	5'-CTGGGAGTGCTGGTGCTGAT-3', 5'-TAACAATCACTCCAAGCAGTATCTC-3'	136–343	208
GSL3	DQ086484	5'-ATTGCCCAAGCGATCAAGCCTGTC-3', 5'-TCTGCTGAACGCCTGGTTGAAAA-3'	246–428	183
GSL6	AM743080	5'-ACGGCGAGACCGACGAAA-3', 5'-GCCAGGAGAAACCGAACCAG-3'	230–451	222
GSL8	DQ086485	5'-TCTATGTGACTACTGTTGGGTT-3', 5'-CACGCTCAATGCTTTATTT-3'	121–275	155
GSL10	DQ086486	5'-GGGGACAGAGCGAGAACA-3', 5'-GTAGGCAACACGAAGAGC-3'	3–195	193
GSL12	DQ086487	5'-GTTCTTCTCGTGGTTCCCCTTT-3', 5'-GTCCCTAATCAAGTCCAGAAATGTA-3'	205–386	182
GSL19	DQ086488	5'-TCATCCCAACTGTCTGGTTTATT-3', 5'-TATCTACGGAGCACAGCCCCACT-3'	282–453	171
GSL22	DQ086488	5'-ATCGTTCTGGGAGTGCTGGTGCT-3', 5'-CCACAGAAATAACACTGCCCCAC-3'	130–307	178
GSL23	DQ086490	5'-AACCTCCGCCTTCCGACAGA-3', 5'-GCATACAGGAACACTCGGAATC-3'	31–222	192

*Primers for both genes were designed according to the conserved region in the corresponding gene orthologs identified different plant species (Dinant *et al.*, 2003) and synthesized against the conserved regions in the *Arabidopsis* orthologs. The primers were used in this study after RT-PCR products obtained from amplifying wheat RNA had been confirmed to be more than 87% identity with the conserved regions of previously reported gene orthologs of different plant species (Dinant *et al.*, 2003; NCBI nucleic acid databases and BLAST searches).

Table S2. Characters of seeds from plants that were not colonized with aphids

Wheat genotype	Long/short axis ratio	Long axis (mm)	Short axis (mm)	Roundness	Equivalent cycle diameter (mm)	Size (mm2)	Circumference (mm)	Weight (g)
Y16	2.15	4.61	2.21	1.27	3.14	7.84	11.57	0.050
Y16:Hpa1$_{10\text{-}42}$#6	2.13	4.58	2.20	1.26	3.11	7.80	11.55	0.049
